# Cereal Coffee

**Published:** 1879-08

**Authors:** 


### CEREAL COFFEE.

[*From The Housekeeper.*]

Warm drinks are a necessity to civilized humanity, and it is of the first importance that we should seek to determine what beverage we shall habitually use. The late Mr. Greeley made it a practice to accompany his breakfast with a cup of hot water, rendered more palatable by the addition of a trifle of milk and sugar. He expressed to us the belief that some hot or quite warm fluid was needed to give tone to the stomach at the morning meal, and to assist the digestive processes. He could not employ tea or coffee without suffering the pangs of indigestion. This is true of multitudes, many of whom are unaware of the reason for their gastric uneasiness. They continue to swallow the very fluid which destroys their comfort, vainly imagining the while, that a stronger dose will give nature an extra jog, and accomplish some benign result. The truth is simply this: one half the human race cannot use an infusion of the tea-plant, nor that of the browned coffee berry, and at the same time maintain reasonably good digestion, and strong, sound nerves. These infusions have a wonderful power to stimulate, and an equal power to depress. Under their influence the brain and nervous system are elevated, exalted, raised, refined. When their short-lived influence ceases, the facility of descent to a lower than the original plane, is startling. It is the brilliant ascent of the rocket, and the dark, and gloomy fall of the stick. It elevates only to narcotize and destroy.

Cocoa and its products have been largely used as substitutes for the well-nigh universal tea and coffee, but they have proved heavy and somewhat difficult of digestion, by thousands. It is a pity that this fragrant bean could not be more generally employed, as it is rich in that element which exists neither in tea nor coffee—we mean nutriment. But the majority declare it to be "sleepy stuff," and although its use is happily extending, it can never become the universal beverage. Probably the cereal grains are the source to which we must go for the perfect food-beverage, in which nutriment and flavor shall be scientifically blended. This is the thought which has actuated the Health Food Company in the preparation of their Cereal Coffee. It is a strictly scientific preparation, compounded of the gluten of wheat and barley. In its manufacture the starch of the cereal is carefully excluded, and only the nitrogenous gluten is employed. The barley portion is carefully browned, so as to impart to the infusion a pleasant, parched flavor. The portion derived from wheat is thoroughly cooked, so that its nutritive qualities are speedily imparted to the infusion. Steeped in a mixture of milk and water, a nourishing fluid results, having a flavor not wholly unlike that of Java coffee, and containing more than ten times as much food-value. If we compare this rich beverage with that obtained from real coffee, we discover that the Cereal Coffee would be worth $3.00 per lb.—equal in nutriment to ten lbs. of old Java at 30 cents per lb. Chicory, and peas, and beans, and rice, and corn and rye, and sweet potatoes, and a host of other substances have been browned and infused in boiling water as substitutes for coffee, but they have all failed, because they have all been based upon starch in various forms. Real coffee contains little starch, and any successful substitute for it must be quite free from that tasteless and nearly useless food-substance. The Cereal Coffee of the Health Food Company appears to fulfill every indication, and to meet the precise want. The beverage produced from it is a powerful supporter of human life, and is an appetising and delicious adjunct to the daily bill of fare, while leaving the brain and nerves uninjured by any noxious stimulating power. Lacking the tannic acid and the powerful narcotizing principle of tea, it neither deranges digestion, induces constipation, nor lowers the vital tone, as does that potent agent for evil. The browning and parching processes being conducted in vaccuo, create no deadly, empyreumatical oil, such as always exists in browned coffee and its [illegible]

